# Believe in You student empowerment program: A pilot study

**DOI:** 10.3389/fspor.2022.1078002

**Published:** 2023-01-16

**Authors:** Erin E. Centeio, Jeanne M. Barcelona, Kevin Mercier, Aaron Hart, John T. Foley

**Affiliations:** ^1^ College of Education, Department of Kinesiology and Rehabilitation Science, University of Hawai‘I at Mānoa, Honolulu, HI, United States; ^2^ College of Education, Division of Kinesiology, Health, and Sport Studies, Wayne State University, Detroit, MI, United States; ^3^ College of Education and Health Sciences, Department of Health and Sport Sciences, Adelphi University, Garden, NY, United States; ^4^ Vice President of Curriculum & Program Engagement, Varsity Brands, Cortland, NY, United States; ^5^ Department of Physical Education, SUNY Cortland, Cortland, NY, United States

**Keywords:** social emotional learning, physical education, COVID-19, youth, CASEL, self-efficacy, self-awareness, self-management

## Abstract

**Introduction:**

The social and emotional health of youth is important, especially after students experience the COVID-19 pandemic. The purpose of this study was to understand the influence that the *Believe In You Student Empowerment Program* had on students social emotional learning (SEL) behaviors over a 10 week period during the COVID-19 pandemic.

**Materials and methods:**

A part of this quantitative study, one school in each the intervention and the control group (delayed intervention; 2 schools total) participated in the study. Students enrolled in physical education within each school participated (*n* = 166; Intervention = 88). Students in each group took a survey at week 1 (baseline measure), week 5, and week 10. Students who were in the intervention group started the program after week 1, while the delayed intervention group began the program in week 5.

**Results:**

A series of ANCOVA's examined the difference of social emotional learning knowledge and social emotional learning scales between the treatment and control groups. Self-awareness (*F* = 13.91, *p* < .01), self-management (*F* = 6.14, *p* < .01) & relationship skills (*F* = 5.50, *p* < .05) saw significant differences over time compared to the control group. The second series of analyses looked only at the intervention group and analyzed to determine significant differences in mean scores of SEL variables between weeks one and ten. Emotional regulation saw significant differences (*t* = 2.5, *p* < .01). The final set of analyses conducted were with the delayed intervention group and examined the difference in mean SEL scores over the three time periods. Again, emotional regulation saw significance with an interaction of time and gender (*F* = 4.162, *p* < .01).

**Discussion and Conclusion:**

In a short period of time, *Believe in You Student Empowerment Program* has shown the potential to have a positive influence on students social emotional learning behaviors, even during the COVID-19 pandemic. More research should be conducted over a longer period of time, in-person, and with an experimental design to better understand the effects of the Varsity Brands *Believe in You Student Empowerment Program* and its implications with student social emotional learning behaviors.

## 1. Introduction

Mental illness among children and adolescents increased significantly during the COVID-19 pandemic (1, 2). Requests for mental health services for children and adolescence increased by as much as 10% (3). Even before COVID, there were increased levels of depression, anxiety and suicide rates among youth (4) which continued to increase substantially throughout the pandemic (5, 6) and disproportionately impacted vulnerable communities (7). Several groups, such as the Centers for Disease Control and Prevention and the U.S. Department of Health and Human Services, identified an urgent need to develop comprehensive strategies to aid youth mental health and identified schools as an ideal place to address these concerns (5, 8).

In addition to public health concerns, COVID-19 brought on additional challenges for teachers regarding their own mental, social, and emotional health, but also in providing the social and emotional support their students needed (9, 10). Sadness, fear, social isolation, and family instability highlighted student concerns during this period (11). Yet, schools persisted and were urged to design safe, supportive, and equitable environments, while strengthening connections to families and communities, aimed at supporting students' social-emotional development (12).

Social and Emotional Learning (SEL) programs have been beneficial in improving the academic performance, healthy relationships, and mental wellness of students (13). SEL programs assist children in gaining knowledge and skills needed to understand and succeed in an ever changing and complex world. The Collaborative for Academic, Social, and Emotional Learning (CASEL) (13) has defined SEL as:

*an integral part of education and human development. SEL is the process through which all young people and adults acquire and apply the knowledge, skills, and attitudes to develop healthy identities, manage emotions and achieve personal and collective goals, feel and show empathy for others, establish and maintain supportive relationships, and make responsible and caring decisions*.

The CASEL framework was established to inform comprehensive development of SEL opportunities that are representative of intrapersonal, interpersonal, and cognitive competence across five domains including: self-awareness, self-management, social awareness, relationship skills and responsible decision making (14). This is important as each domain targets specific aspects integral to the development of a socially competent individual. The CASEL framework pinpoints three contexts where SEL is critical; the *classroom, schools, and in the home and communities*. Further, the framework indicates that effective SEL approaches are: Sequenced, Active, Focused, and Explicit (SAFE) suggesting that SEL should be taught intentionally and through targeted curriculum that can empower youth personally and socially through coordinated and engaging activities (14). As such, the CASEL framework is an effective tool that educators can utilize to guide the development of SEL curriculum critically needed by schools, teachers and students in the ever-evolving mental health crisis's that have been exacerbated by the COVID-19 pandemic.

Given the importance that SEL plays in the lives of youth, this study sought to address students' social emotional health through the *Believe in You Student Empowerment Program (Believe in You)*.

### 1.1. Theoretical underpinnings of the believe in you program

Empowerment Theory and the SEL framework for CASEL (14) were overarching frameworks for the original program design of *Believe in You* and also guided this evaluation and pilot study. Broadly, Empowerment Theory suggests that the degree to which someone feels empowered is established through a series of experiences and related outcomes (15). Specifically, the theory posits that personal empowerment is influenced by the actions, activities and structures in which an individual engages and that the outcome of their individual experience is the level of empowerment that they perceive (15). Guided by the understanding that empowerment is an outcome of the experiential processes an individual is exposed to, Varsity Brands created a program designed to empower students by offering safe, empowering and healing experiences through which they can learn the basis and application of core social and emotional competencies. In doing so, the goal of the program was to create a process through which students were provided opportunities to become stronger and more confident, especially in controlling their life and claiming their rights.

**Figure 1:**
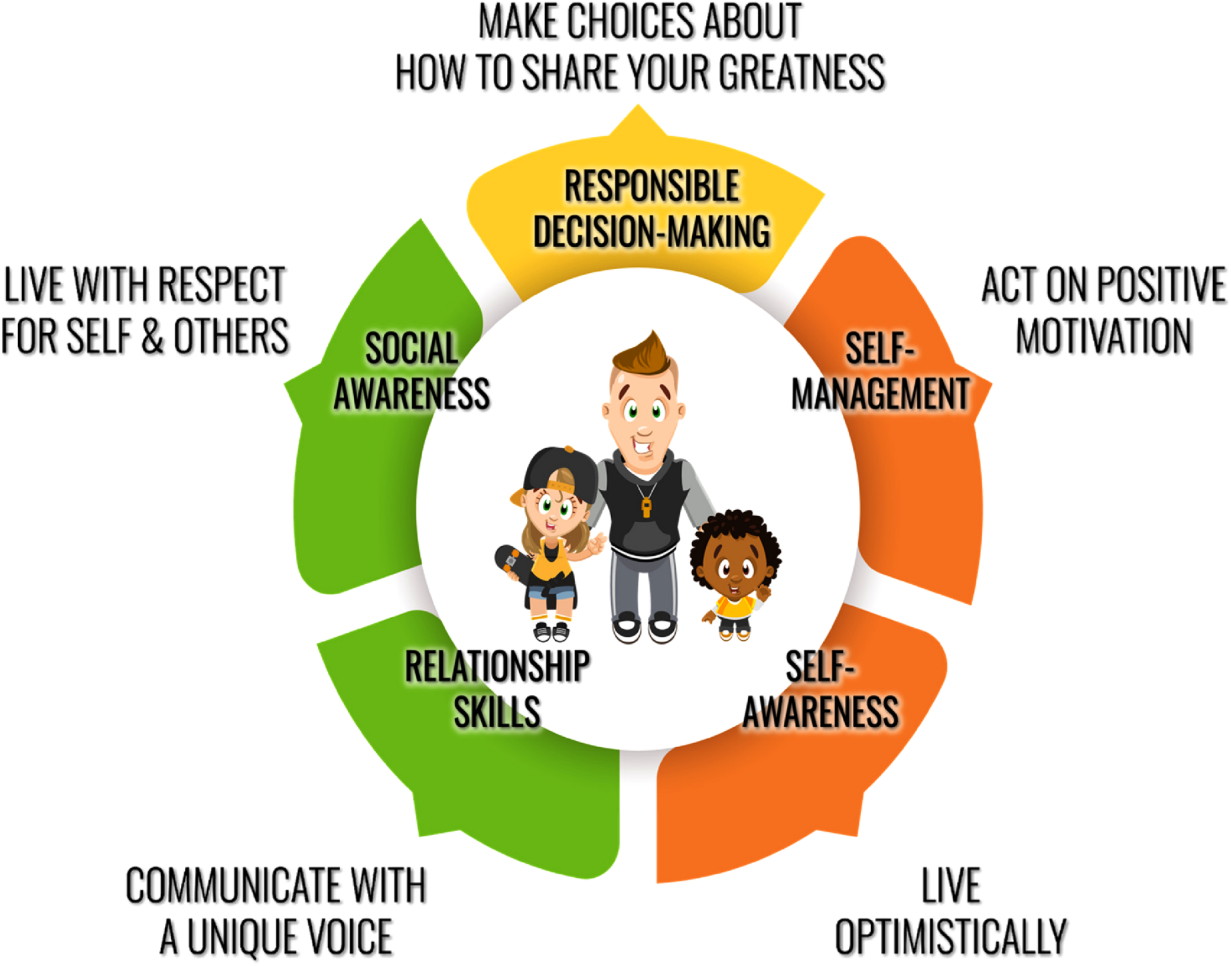
*Believe in You* empowerment rights. Used with permission from Varsity Brands.

The development of the *Varsity Brands Student Empowerment Rights* was informed by Empowerment Theory and CASEL core competencies. Empowerment rights were first developed as a foundational aspect of the program to enhance students' opportunity to, “discover, or create and give voice to, a collective narrative that sustains their own personal life story in positive ways.” (16) These rights were written to serve as educational programming outcomes aligned to the CASEL core SEL Competencies of self-awareness, self-management, social awareness, relationship skills, and responsible decision-making (13). *Varsity Brands Student Empowerment Rights* framework can be seen in Figure 1 and shows that all students have the right to: (1) Live optimistically (aligned with self-awareness CASEL competency), (2) Act on positive motivation (aligned with self-management CASEL competency), (3) Live with respect for self and others (aligned with relationship skills CASEL competency), (4) Communicate with a unique voice (aligned with social awareness CASEL competency), and (5) Make choices about how to share their greatness (aligned with responsible decision making CASEL competency).

*Varsity Brands Student Empowerment Rights* is the basis of the *Believe in You* program which encompasses SEL and uses journaling prompts based on research that suggests positive effects of journaling can help control anxiety and improve mental and emotional health for both students and adults (17, 18). The journals include weekly prompts that work toward one of the five empowerment rights while also introducing emotional vocabulary words through questions and prompts that help students relate SEL concepts back to their personal lives. The journaling format provides the space needed for students to create and tell their story with the help of the positive language and SEL guided discovery.

Therefore, the purpose of this pilot study was to understand the influence that the *Believe in You* had on students SEL behaviors and knowledge over a 10-week period during the COVID-19 pandemic.

## 2. Methods

After IRB approval, six districts were recruited to participate in a quasi-experimental designed intervention and subsequent program evaluation. This study was deemed as exempt through the IRB process as it was specifically interested in conducting program evaluation. Although exempt, all districts sent home an opt-out form that allowed parents and gaurdians the ability to opt out their child from participation in the research/evaluation portion of the study. However, because the Belive in You Curriculum was adopted by the schools, all youth participated in the program as part of their normal daily curriculum. Due to COVID-19 and corresponding issues with online learning only three of the six districts recruited agreed to participate. One district consisting of three schools and located in the Southeast region of the U.S., was assigned as the intervention group and two districts, located in the Southwest and Midwest regions, representing a total of two schools served as the comparison group and received delayed intervention. As such, a total of three districts representing five schools participated at baseline (T1). Across the participating districts, a total of four physical education teachers participated in the study facilitating programming across the classrooms/gyms they taught as well as reporting program implementation on a weekly basis to ensure fidelity. In the intervention group, one teacher taught at each of the three intervention schools as the primary physical education teacher. In the delayed intervention group, one teacher taught at one school, and two teachers taught and implemented the program in the other school. For the purpose of this evaluation, the term “classroom” will be used to represent the teachers within the school and the students that they taught.

The schools participating in this study were provided journals and professional development free of charge through the support of Varsity Brands. They were invited into the study based on teacher and administrative cooperation and an ability to submit student data and implementation details. The evaluation team prioritized schools that served a significant number of students from low-income families, as the schools voiced students may not otherwise have access to social and emotional support during the COVID-19 pandemic.

### 2.1. Program overview

The *Believe in You* program is designed to provide students with exposure to SEL concepts by presenting them with journaling prompts. Each journal prompt aims to facilitate self-exploration and encourages students to process their thoughts and experiences through the development and utilization of self-awareness, self-management, social awareness, relationship skills and responsible decision-making. For example, “In your own words, what does it mean to be trustworthy?” or “This week we have explored trust and optimism. Think about the things that make you feel trust. Write a few of those things in the “causes” box to the right.”

The main programmatic feature is the *Believe in You* Empowerment SEL journals, which is a 40-week student journal that includes 20 different social and emotional learning vocabulary words. As described above, the program was designed to align with CASEL's five competencies of SEL and content is scaffolded so that in the first 10 weeks, students are first introduced to the social and emotional concepts that are then revisited and built upon over the course of the 40 week program. The journals provide daily, specific writing prompts designed to be safe spaces for students to process their thoughts and discover their emotions. Teachers are encouraged to use journal prompts for class discussions and/or have students submit weekly writing samples. Although the program is designed to be flexible and allow schools and teachers to use it as they see fit within their school structure, it is common for teachers to take the first 5–10 min of class and have students write in their journals as “bell-work”. Of course, different teachers enact it differently depending on their schedule and how they see it best fitting into their school day.

Programmatic enhancements include *Believe in You* weekly announcements and SEL academic vocabulary posters. The *Believe in You* video series were also developed to enrich the overall student user experience. The videos range between 8 and 10 min and are designed to coincide SEL competencies that the students learn about. There are currently four seasons of videos, with a total of 7–9 videos in a season. Teachers are asked to use them as they deem necessary and although they come with separate lesson plans, they are considered supplementary and therefore not prescribed within the *Believe In You* program where to have students' watch individual lessons. All of these materials are available for download free of charge at BelieveInYou.com. Print version of the student journals were provided free to the schools participating in this study.

#### 2.1.1. Study implementation

The schools that participated in this study took advantage of all of these resources and reported the regular use of empowerment journals, class discussion, collection of writing samples, and the *Believe in You* video series on a weekly basis.

Although *Believe in You* can be implemented in any part of the school day or within a before or after school program: however, all participating schools in this study implemented programming during physical education class. Given the evaluation took place during the COVID-19 pandemic “normal” physical education classes were not the norm. All students in the study did participate in physical education in person. On average teachers reported seeing their students for a single class period (30–50 min), ranging from 2 to 4 days per week (Mean = 3.74 days). In alignment with the reported time students spent in their physical education class, teachers also indicated they utilized the *Believe in You* resources each class session reporting that students wrote in their journals an average of 3.63 days per week and the inclusion of *Believe in You* programmatic enhancements included in class instruction 3.26 days per week.

#### 2.1.2. Professional development overview

Professional development was provided to the schools participating in this study and it was standardized for both the intervention and delayed intervention groups. Intervention and delayed intervention groups received the standardized training from the same facilitator *via* Zoom, five weeks apart. The facilitator has New York State permanent certification for K-12 health and physical education with a Master's of Science in Education, and is also the author of the *Believe in You*.

The professional development provided was 90 min in duration and the agenda included: introduction and overview of the CASEL 5 Core SEL Competencies as well as detailed overview of the program design and alignment to the CASEL 5 Core Competencies. First, program alignment was described in relationship to the *Believe in You* Student Empowerment Rights. Trauma Informed Pedagogy was also introduced within the context of establishing safe learning environments, providing empowering support, and maintaining a safe and empowering environment consistently for students' overall social and emotional wellbeing.

Next, each component of the program was introduced with a focus on the structure and design of the student journals. Teachers were introduced to weekly social and emotional learning class announcements, academic vocabulary resources, and depth of knowledge question scaffolded question stems. Teachers were given guidance and strategies for how to use journal prompts to facilitate discussion *via* community circles including discussion around solutions for virtual learning. Additionally, teachers were walked through all of the free resources online and explained how to utilize them with the student journals.

Lastly, teachers were given instruction on how to facilitate student surveys and report implementation details. A 15-minute question and answer period was provided.

### 2.2. Participants

#### 2.2.1. School demographic information

##### 2.2.1.1. Group 1 schools

###### 2.2.1.1.1. District #1—school #1 (grade 9)

Student demographics: 61% White, 17% Black, 15% Hispanic, 2% Asian/Pacific Islander; 59% of students come from low-income families; Reading proficiency 70%; Enrollment 82 students.

###### 2.2.1.1.2. District #1—school #2 (grades 6–12)

Student demographics: 33% White, 26% Black, 25% Hispanic, 9% Asian/Pacific Islander; 97% of students come from low-income families; Reading proficiency 40%; Enrollment 117 students.

###### 2.2.1.1.3. District #1—school #3 (grades 9–12)

Student demographics: 66% White, 12% Black, 12% Hispanic, 7% Asian/Pacific Islander; 35% Students from low-income families; Reading proficiency 85%; Enrollment 233 students.

##### 2.2.1.2. Group 2 schools

###### 2.2.1.2.1. District #2—school #1 (grades 9–12)

Student demographics: 8% White, 8% Black, 70% Hispanic, 8% Native American; 98% of students come from low-income families; Reading Proficiency 19%; Enrollment 1,128 students.

##### 2.2.1.3. Group 3 schools

###### 2.2.1.2.2. District #3—school #1 (grades 7 and 8)

Student demographics: 8% white, 75% Black, 13% Hispanic, 2% Asian/Pacific Islander; 100% students from low-income families; Reading Proficiency 17%; Enrollment 796 students.

District #1—School #2 (Grades 6–12)

#### 2.2.2. Student demographic information

At each school, participating physical education teachers taught the *Believe in You* lessons as a portion of their regularly scheduled classroom sessions and encouraged their students to participate in the survey as part of their regular class assignments. Therefore, the total amount of students enrolled in physical education (202 students; intervention = 104; comparison = 98) within each teacher's class participated in the study. District #1(3 schools) had a total of 105 students, district two (one school) had 83 students, and district three (one school) had 14 students. At T1, of the total student sample, 186 (intervention = 97; comparison = 89) completed it. Overall participants had a mean age of 14.82, with a range from 13 to 18 years old. The group overall reported being 37% male, 60% female, and 3% other. The intervention group had a mean age of 15.1 years (60% female), while the delayed intervention group had a mean age of 14.48 years (58.6% female). No race/ethnicity variables were collected during this program evaluation.

### 2.3 Data collection

Student level data were collected at three time points. Students in the intervention group were administered a survey at week 1 (baseline measure; T1), week 5 (T2), and week 10 (T3). Students who were in the intervention group started the program after week 1. Students in the delayed intervention group completed both the T1 measure, and the T2 measure at the 5-week mark, in order to serve as a delayed intervention group, before starting the *Believe in You* program. The delayed intervention group participated in five weeks of the program before completing the third survey (T3). Although there were limitations to the comparison approach utilized, COVID-19 related barriers, as well as the desire for schools to implement the program with their students, led to this as the best design that could accommodate all students. Data for T1 were collected between February 1st and February 26th, 2021, data for T2 were collected between March 15th and April 9th, 2021, and T3 data were collected between the weeks of May 17th to June 1st, 2021.

### 2.4. Instruments

In alignment with the core components of the CASEL framework that guided the development of the *Believe in You* program, a survey instrument consisting of a total of 49 questions was developed to assess demographics, SEL knowledge as well as outcomes of social and emotional competency including emotion regulation, self-management behaviors and self-efficacy. Demographic questions were only completed about school, teacher, age, and grade. A total of 23 knowledge questions were also included and covered the five CASEL SEL competencies [self-awareness (SA), self-management (SM), social awareness (SOA), responsible decision making (DM), and relationship skills (RS)]. In addition to the knowledge questions, three previously validated scales, that totaled 21 additional questions, were used to measure students' emotional regulation (ER), self-management (SMS), and self-efficacy (SE) (19). These scales were previously validated and are part of the Panorama battery of SEL items. Further descriptions of each can be found below. Given the students were in-person, the surveys were administered in two ways. Either they were read out loud by the teacher and students completed as they were read, or students took the surveys in a computer lab and the teacher asked the students to raise their hand for help if they didn't understand a question. The among of help given to the students was determined by their physical education teacher who had a good handle on their language and reading abilities.

#### 2.4.1. SEL knowledge

To better understand how the Believe in You program influenced students' social and emotional knowledge, the survey included 23 knowledge questions that covered the 5 CASEL SEL competencies [self-awareness (SA), self-management (SM), social awareness (SOA), responsible decision making (DM), and relationship skills (RS)]. The knowledge questions were initially created by two evaluation experts based on the 10-week *Believe in You* program. After item development, two outside researchers/evaluators and one content expert examined the questions for clarity. Finally, two middle school children (*n* = 2) piloted the questions to provide additional clarity and understanding. Although it was not an ideal nor complete pilot, it was all the researchers had access to within a limited period of time. One example of a knowledge question was “Fill in the blanks. People who act with courage feel _______first, but they do what's right, even if they are feeling _________.”

#### 2.4.2. Emotional regulation (ER)

The emotional regulation scale, which consisted of six questions that were based on a 5-point Likert scale, was used to investigate changes in SEL-based competency. The choices on the 5-point scale differed depending on the question. One example of a question is “When you are feeling pressured, how easily can you stay in control?” and it ranged from *not easily at all* (1) to *extremely easily* (5). Once data was reverse coded (if needed) and cleaned, reliability analysis was conducted to ensure reliability within the scale. Cronbach's alpha was calculated at each time point (T1, *α* = 0.82; T2, *α* = 0.84; T3, *α* = 0.82) affirming reliability.

#### 2.4.3. Self-management (SM)

The self-management scale, which consisted of 10-items, was also used to investigate changes in “acting on positive motivation”, which is aligned with the empowerment rights framework. It asked students “in the last 30 days….” and requested a response on a 5-point Likert scale ranging from *almost never* (1) to *almost all of the time* (5). An example of a question is “How often do you come to class prepared?” Reverse coding for questions happened before analysis. Once data was reverse coded (if needed) and cleaned, reliability analysis was conducted to ensure reliability within the scale. Cronbach's alpha was calculated at each time point (T1, *α* = 0.79; T2, *α* = 0.85; T3, *α* = 0.80) affirming reliability.

#### 2.4.4. Self-Efficacy (SE)

The self-efficacy scale, created by Panorama Education, consisted of five questions that had students answer on a 5-point Likert scale. The options in the scale ranged from *not confident at all* (1) to *extremely confident* (5). An example question is “How confident are you that you can complete all the work that is assigned in your classes?” Once data was reverse coded (if needed) and cleaned, reliability analysis was conducted to ensure reliability within the scale. Cronbach's alpha was calculated at each time point (T1, *α* = 0.87; T2, *α* = 0.86; T3, *α* = 0.83) affirming reliability.

### 2.5. Data analysis

A series of quantitative analyses were conducted to determine significant differences of five SEL knowledge variables and three SEL scales over time. First, a series of independent *t*-tests were run to determine differences at baseline among the intervention and delayed intervention group. Then a series of ANCOVA's were conducted, controlling for time 1 variables, age, and gender, to determine differences between the intervention and control groups from weeks one and five. Then a series of paired sample *t*-tests were run to determine differences in the intervention group from weeks one through ten.

## 3. Results

### 3.1. Baseline differences

A series of independent *t*-tests were run to determine if group differences were present among the outcome variables. Results showed that there were significant differences at baseline among the intervention and delayed intervention groups for SEL knowledge (*t* = 5.89, *p* < .001) and self-management (*t* = 5.60, *p* < .01). There were no significant differences between groups in relation to self-efficacy (*t* = −0.21, *p* > .05) and emotional regulation (*t* = 0.53, *p* > .05).

### 3.2. ANCOVA results

Four separate ANCOVA's were conducted with T2 (of given variable) as the dependent variable, group as the independent variable, and T1 (of given variable), grade level, teacher, and gender as covariates. Outcome or dependent variables included SEL knowledge, self-efficacy, self-management, and emotional regulation.

Overall results showed that students SEL knowledge, emotional regulation, and self-efficacy were all significantly higher at T2 within the treatment group when controlling for T1, gender, age, and teacher. Self-management was trending towards significance with a *p* value of *p* < .059. ANCOVA details are noted below.

#### 3.2.1. SEL knowledge

The first ANCOVA examined the difference in groups (comparison and intervention) of total SEL knowledge at T2 (which included self-awareness, self-management, social awareness, responsible decision making, and relationship skills), while controlling for T1 SEL knowledge, gender, grade, and classroom. Results showed a significant overall main effect of SEL Knowledge F(1,5) = 33.11, *p* < .001, *η*2 = 0.51, Adj. *R*^2^ = 0.49. T1 SEL knowledge F(1,5) = 87.84, *p* < .001, *η*2 = 0.36, and group F(1,5) = 3.73, *p* = .05, *η*2 = 0.02 had a near significant effect, while gender, age and classroom were non-significant (*p* > .05). The mean for SEL knowledge at T2 was significantly higher among the treatment group (17.7) than the delayed intervention group (14.6).

#### 3.2.2. Emotional regulation

The second ANCOVA examined the difference in groups (comparison and intervention) of emotional regulation at T2 while controlling for T1 emotional regulation, gender, grade, and classroom. Results showed a significant overall main effect of emotional regulation F(1,5) = 37.25, *p* < .001, *η*2 = 0.54, Adj. *R*^2^ = 0.53. T1 emotional regulation F(1,5) = 154.97, *p* < .001, *η*2 = 0.49, and group F(1,5) = 6.21, *p* < .01, *η*2 = 0.04 had a significant effect, while gender, age and classroom were non-significant (*p* > .05). The mean for emotional regulation at T2 was significantly higher among the treatment group (3.5) than the delayed intervention group (3.2).

#### 3.2.3. Self-management

The third ANCOVA examined the difference in groups (comparison and intervention) of self-management at T2 while controlling for T1 self-management, gender, grade, and classroom. Results showed a significant overall main effect of self-management F(1,5) = 46.43, *p* < .001, *η*2 = 0.61, Adj. *R*^2^ = 0.59. T1 self-management F(1,5) = 177.261, *p* < .001, *η*2 = 0.55 was significant, while group F(1,5) = 3.69, *p* = .059, *η*2 = 0.03 had a near significant effect. Gender, age and classroom were non-significant (*p* > .05). The mean for self-management at T2 was significantly higher among the treatment group (4.1) than the delayed intervention group (3.7).

#### 3.2.4. Self-efficacy

The fourth ANCOVA examined the difference in groups (comparison and intervention) of self-efficacy at T2 while controlling for T1 self-efficacy, gender, grade, and classroom. Results showed a significant overall main effect of self-efficacy F(1,5) = 36.69, *p* < .001, *η*2 = 0.54, Adj. *R*^2^ = 0.52. T1 self-efficacy F(1,5) = 163.61, *p* < .001, *η*2 = 0.51, and group F(1,5) = 4.05, *p* < .05, *η*2 = 0.03 had a significant effect, while gender, age and teacher were non-significant (*p* > .05). The mean for self-efficacy at T2 was significantly higher among the treatment group (3.6) than the delayed intervention group (3.2).

### 3.3. Paired sample *T*-tests results intervention group

Table 1 represents *t*-test results indicating changes from baseline (T1) to week ten (T3) for the intervention group. In general, not taking into account a delayed intervention group, over the 10-week program students significantly increased their efficacy and emotional regulation. Although not statistically significant, improvements in self-management and SEL knowledge can be seen and have medium (self-management) and large (SEL knowledge) effect sizes indicating that the lack of significance could be due to the small sample.

**Table 1:** *T*-test results indicating changes from baseline (T1) to week ten (T3) for the intervention group.

Variable	Mean	Std. Deviation	95% confidence interval		*t*-value	*p*-value	Cohen's d
SEL Knowledge	0.37	2.52	−0.36	1.09	1.02	0.31	2.54
Emotional Regulation*	0.40	0.63	0.22	0.58	4.44	0.00	0.63
Self-Management	0.067	0.42	−0.05	0.19	1.13	0.26	0.42
Self-Efficacy*	0.36	0.65	0.17	0.54	3.83	0.00	0.65

* Indicates statistical significance at *p* < .001.

## 4. Discussion and conclusions

The COVID-19 pandemic led to increased levels of depression, anxiety and suicide rates among youth (6). Schools were called upon to implement SEL programs aimed at supporting students' social-emotional development (12). The *Believe in You* program is one such developed to address growing concerns among children and adolescents well-being. Over the period of ten weeks, secondary students across three school districts, representing five schools participated in *Believe in You* program. The results of the study showed that students who were part of the intervention group, significantly increased their SEL knowledge, efficacy, and emotional regulation above and beyond the delayed intervention group over the five weeks of program implementation. Furthermore, this trend continued through 10 weeks of the program with students continuing to significantly increase emotional regulation and self-efficacy. The intervention group results are promising and suggest that when students are provided the *Believe in You* journals and supported through the program, they continue to hone their social and emotional skill sets. It is also important to note that after receiving programming beginning at the five week mark, the delayed intervention group exhibited significant changes for student's efficacy. This is promising because it indicates that when utilizing the *Believe in You* program and journals, students' can begin to improve their self-efficacy in a very short amount of time. Further, given that self-efficacy is an important precursor to social and emotional growth these results suggest an ideal progression of the skill sets gained while utilizing the *Believe in You* program.

The results of this study also extend to the current and ever-evolving COVID-19 context and its implications for student social and emotional health as a result. A review of the literature on the effects of COVID-19 pandemic on mental health of children and adolescents, indicates that there was a substantial impact on child and adolescent anxiety and depression (20). Many speculate that some of this anxiety may have been related to changes in the learning environment during the pandemic and the fact that there was a lot of uncertainty within school districts not knowing if classes would continue remotely or move to in-person instruction, which in turn led to other worries about basic needs being met (21). Further, educators had to shift their pedagogical approaches as well as the curricular objectives and educational standards they addressed. For example, Mercier and colleagues (22) indicated that the majority of physical educators reported decreased instruction time and therefore prioritized getting students to increase their knowledge in health-related fitness and/or enjoy physical activity during the COVID-19 pandemic (SHAPE standards 3 & 5 respectively), leaving little time to engage students in SEL based instruction. Other research has discussed the importance of teachers acknowledging the struggles and trauma that students experienced as part of the COVID-19 pandemic and the importance to ensure the priority of mental health among students moving forward (23). As such, the *Believe in You* program is timely and warranted as schools and districts worldwide look for feasible and effective ways to assist students as they work to overcome the ongoing traumas resulting from the COVID-19 pandemic.

Despite the positive and compelling findings of this study, it is not without limitations. There were many challenges due to the COVID-19 pandemic which impacted the design of the intervention, because it created interruptions in class time and intermittent changes in instruction. However, students still saw positive SEL gains over a short period of time, while controlling for important variables such as classroom, age, and gender. This has positive implications for the 40-week *Believe in You* program because it suggests that educators can expect to see immediate and positive improvements in their students social and emotional growth, especially as teachers and students navigate the on-going uncertainties of the COVID-19 pandemic. In addition to COVID-19 challenges, there were other limitations that need to be acknowledged within the study. First, the sample size for this study was also small and our instruments did not collect race and ethnicity at the student level. This is unfortunate given recent research indicating that with COVID-19, youth needs vary greatly across racial and ethnic groups. Taken collectively, the small sample size and lack of information on race and ethnicity may suggest that results cannot be generalized to the larger population. However, given the positive and significant findings indicated in this study, we anticipate that future studies with a larger, diverse sample may yield more significant results. Another limitation to this study was the difference of reading proficiency at each of the schools. Given difficulties with the COVID-19 pandemic, the researchers could not provide a controlled match which could have influenced the overall results. Third, given that during the time of data collection, researchers were still not being allowed physically in schools, it was hard to properly pilot with a like population the full survey. A convenience sample of two middle school students was used, but is an acknowledged limitation and the readers should interpret how they see fit. Finally, the current study was only able to evaluate the impact of 10 weeks of programming, thus not capturing longitudinal changes over the full 40 week program. Regardless of this limitation, we know that student level changes are seen within a 10-week period suggesting that additional time engaging with the entire 40 week *Believe in You* program has the capacity to substantially increase knowledge and substantially support their acquisition of social and emotional skills that are vital for school, work, and lifelong success.

In conclusion, this study suggests that the *Believe in You* program is a feasible and effective way to integrate SEL into physical education and school setting. Future studies should look to longitudinally evaluate the impact of the *Believe in You* program within a larger sample. Further, it will be important to explore how racial and ethnic variations in program impact as this can better inform how we differentiate our instructional approaches for SEL-based learning. Additionally, future research should seek to better understand how SEL based programs such as *Believe in You* are implemented by physical educators and classroom teachers. Finally, future research should also seek to identify best practices for integrating SEL based programming into the physical education and classroom settings, especially as we emerge from the ongoing COVID-19 pandemic.

## Data Availability

The original contributions presented in the study are included in the article/Supplementary Materials, further inquiries can be directed to the corresponding author/s.
